# The Role of FOXP3 on Tumor Metastasis and Its Interaction with Traditional Chinese Medicine

**DOI:** 10.3390/molecules27196706

**Published:** 2022-10-08

**Authors:** Benxu Ma, Wenjun Miao, Jieqiong Xiao, Xinyi Chen, Jing Xu, Yinan Li

**Affiliations:** 1 Affiliated Qingdao Central Hospital, Qingdao University, Qingdao 266000, China; 2 College of Chemistry and Pharmarceutical Sciences, Qingdao Agricutural University, Qingdao 266000, China

**Keywords:** FOXP3, malignant tumor, metastasis, TCM

## Abstract

Forkhead box protein 3 (FOXP3) is an important transcription factor for regulatory T cells (Tregs) and plays an important role in their immunosuppressive function. In recent years, studies have found that FOXP3 is expressed in many kinds of tumors and plays different roles in tumors’ biological behaviors, including tumor proliferation, metastasis, drug resistance, and prognosis. However, the effects of FOXP3 on tumor metastasis and its interaction with traditional Chinese medicine (TCM) remain unclear. Therefore, in this review, we focus on the effects of FOXP3 on tumor metastasis and its relationship with TCM, which can provide evidence for further research and therapy in clinical settings.

## 1. Introduction

**Figure 1:**
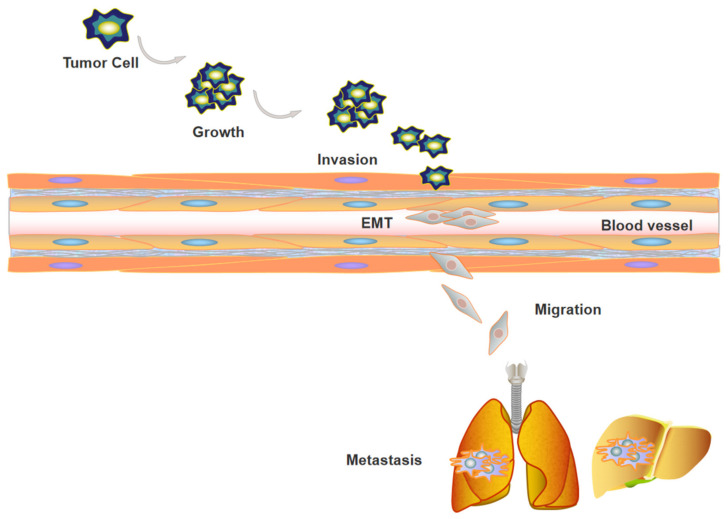
Tumor metastasis contains a series of events. First, clonal expansion of tumor cells was observed, and tumor cells adhere to and invade into basement membrane, during this process, tumor cells proceed EMT. Subsequently, tumor cells extravasate to blood vessels, migrate to distant organs, and form metastatic tumors eventually.

Tumor metastasis is a multistep process involving several events, including tumor angiogenesis, invasion, epithelial–mesenchymal transformation (EMT), tumor immune escape, and the abnormal activation of signal transduction pathways [1,2,3,4,5] (Figure 1). A variety of transcription factors, chemokines, coding/noncoding RNAs, etc., participate in the above-mentioned tumor biological events. At present, the main therapies for tumor metastasis include inhibiting angiogenesis, preventing EMT, and searching for effective metastasis or immunocheckpoint inhibitors. Moreover, traditional Chinese medicine (TCM) has been used as a supplementary or alternative therapy for tumor metastasis. The complexity of the tumor metastasis process demands effective interventions. Thus, new targets involved in the regulation of tumor metastasis need to be found.

FOXP3 (forkhead box protein 3) is an important member of the forkhead/pterygoid helix transcription factor family and functions as both a transcriptional activator and a repressor [6,7]. FOXP3 was originally identified for its mutation that caused lethal autoimmune diseases in mice and humans [8,9]. FOXP3 is localized in the nucleus and highly expressed in Tregs. It is considered as a marker molecule of CD4^+^CD25^+^ Tregs and plays a crucial role in their development and immunosuppressive function [10]. Subsequently, several studies have shown that FOXP3 is also expressed in a series of tumors and influences tumor metastasis by regulating angiogenesis, invasion, and EMT through the abnormal activation of certain signaling pathways [11,12,13].

This review focuses on the signaling pathways of FOXP3 in different tumor metastasis processes and clarifies the interaction between anti-tumor TCM and FOXP3, as well as its potential therapeutic effects.

## 2. General Properties of FOXP3

**Figure 2:**
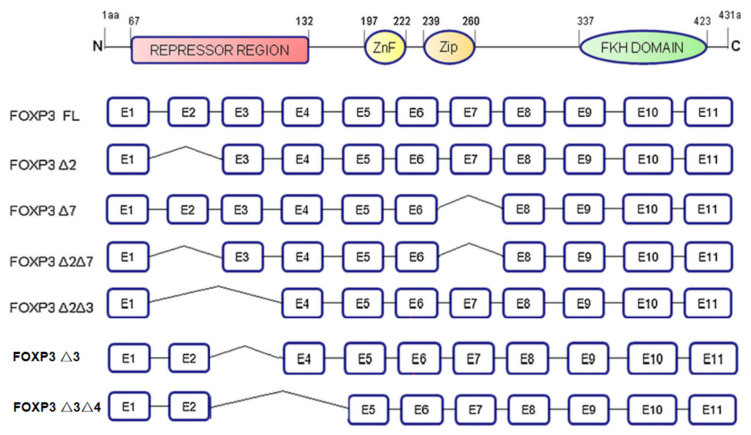
The structure of FOXP3 and its isoforms. FOXP3 gene contains 4 domains, including N-terminal domain, zinc-finger motif, leucine-zipper motif, and FKH domain. FOXP3 is classified into seven different isoforms based on different exons deletion.

The FOXP3 gene is located at Xp11.23. It is a gene whose mutation leads to X-linked autoimmune diseases in mice and humans and is known as a specific marker of Tregs. The FOXP3 gene contains 11 coding exons and 3 noncoding introns. It includes the following: The N-terminal domain (1–132 amino acids), which contains a proline-rich repressor domain; the zinc-finger motif (197–222 amino acids); a leucine-zipper motif (239–260 amino acids), which promotes gene homo- and heterodimerization; and a C-terminal forkhead (FKH) domain (337–423 amino acids), which is a highly conserved DNA-binding site. FOXP3 encodes a variety of mRNA isoforms [14], including full-length FOXP3 (FOXP3 FL), the exon 3 deletion isoform (FOXP3-Δ3), the exon 2 deletion isoform (FOXP3-Δ2), the exon 3 and exon 4 deletion isoform (FOXP3-Δ3Δ4), the exon 7 deletion isoform (FOXP3-Δ7), the exon 2 and exon 7 deletion isoform (FOXP3-Δ2Δ7) and exon 2 and exon 3 deletion isoform (FOXP3-Δ2Δ3). These different isoforms, arising from alternative splicing, are expressed in different cell lines or tumor tissues and play different roles (Figure 2).

Researchers have found that FOXP3 is expressed in a variety of tumors and is closely related to the occurrence, development, and prognosis of tumors over recent years [15].

## 3. FOXP3’s Effects on Tumor Metastasis

### 3.1. Digestive System Tumor

#### 3.1.1. Hepatocellular Carcinoma (HCC)

**Figure 3:**
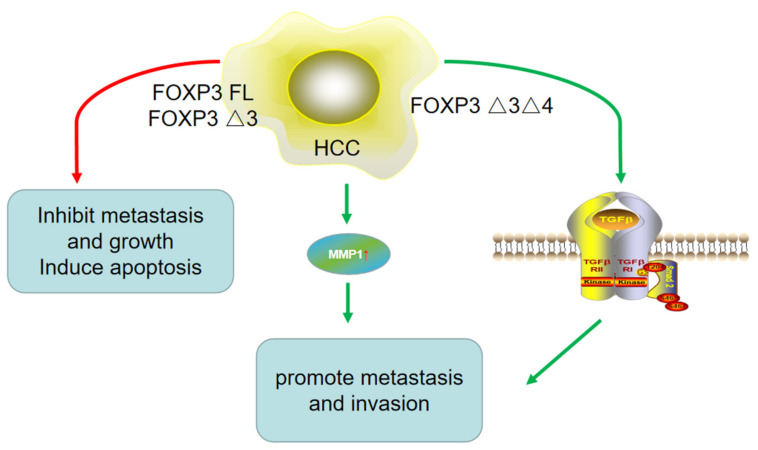
The role of different isoforms of FOXP3 in HCC metastasis. On the one hand, FOXP3 FL and FOXP3-Δ3 inhibited HCC metastasis, while on the other hand, FOXP3-Δ3Δ4 promoted HCC metastasis and invasion by upregulating MMP1 expression and TGF-β/Smad2/3 pathway. It suggested different isoforms of FOXP3 might play contrary roles in HCC.

The role of FOXP3 in HCC metastasis is controversial (Figure 3). It is believed by some that FOXP3 inhibits tumor metastasis and growth, and induces apoptosis. FOXP3 FL and FOXP3-Δ3 have been shown to be expressed in HCC tissues, cell lines, and the mice tumor model. In addition, co-immunoprecipitation results have further revealed molecular mechanisms for the role of FOXP3 in tumor proliferation and apoptosis, and an in vivo studies suggested that FOXP3 can inhibit tumor growth by suppressing c-Myc directly or by interacting with the Smad2/3/4 pathway. However, the mechanism of the effect of FOXP3 in HCC metastasis has not yet been explored [16] (Table 1).

On the other hand, work by other groups shows contrary findings. While Shi et al. [21] found that FOXP3 could function as a tumor suppressor gene and play an antimetastatic role in HCC, they also found that the distant metastasis rate in HCC patients with a high level of FOXP3 was actually remarkably higher than that in negative patients. FOXP3 was constitutively expressed in HCC cells as the FOXP3-Δ3Δ4 isoform. In vitro studies have shown that the expression level of FOXP3 increases with the enhancement of the metastatic potential of tumor cell lines. In this mechanism, FOXP3 likely exerts its inhibitory effects on tumor metastasis by regulating the TGF-β/Smad2/3 pathway. In addition, clinical studies have demonstrated that a high level of FOXP3 expression is correlated with low-level AFP, non-vascular infiltration, and the TNM stage, and FOXP3 expression is also positively correlated with long-term survival and low recurrence rates. Furthermore, a high level of FOXP3 in HCC cell lines could promote metastasis and invasion by regulating the expression of MMP1 (matrix metalloproteinase 1). It can serve as an independent prognostic biomarker for patients with HCC [22]. These contrary conclusions indicate that FOXP3 plays different roles in HCC, which might be related to the different FOXP3 isoforms expressed in HCC (Table 2).

#### 3.1.2. Cholangiocarcinoma (CCA)

In CCA, FOXP3 expression is significantly higher than that in pericarcinomatous tissues. Furthermore, a high level of FOXP3 is positively related to lymph-node metastasis. A study of the molecular mechanism showed that FOXP3 promotes tumor metastasis and invasion might be associated with the upregulation of MMP2 and MMP9 expression. Although this study also found that patients with low levels of FOXP3 had slightly higher median survival, there was no statistically significant difference [19] (Table 2).

#### 3.1.3. Gastric Cancer (GC)

The effect of FOXP3 on tumor metastasis in GC is still unclear.

Some researchers have found that FOXP3 promotes metastasis in gastric cancer. The expression of FOXP3 in 15 tumor tissues of GC patients was obviously higher than that in normal tissues. Interestingly, FOXP3 localization was found to be different across cell types. FOXP3 was expressed in both the nucleus and cytoplasm of tumors. It was mainly expressed in the cytoplasm of normal tissues, and FOXP3 promoted tumor migration, invasion, and proliferation by accelerating the secretion of TGF-β [20]. Besides, Cui et al. [21] found that FOXP3 is the transcription factor of long non-coding RNA (IncRNA) WFDC21P, which could activate the transcription of this IncRNA directly. Moreover, WFDC21P promoted GC cell line proliferation, invasion, and metastasis by regulating the activity of the Akt/GSK3β/β-catenin signal pathway in vitro and stimulated GC metastasis to the lung in vivo. Moreover, another retrospective analysis of 60 cases of GC patients found that the expression of FOXP3, HER2/neu, and Ki-67 was positively associated with the TNM stage and perineural invasion. The data suggest that high FOXP3 and HER2/neu expression and a high proliferation index are, together, a biomarker of tumor aggressiveness [22]. According to Wang et al. [23], miR-664a-3p enhanced invasion, migration, and EMT (upregulating the markers E-cadherin, downregulaing N-cadherin, Vimentin, Sail, and Slug) in vitro and in vivo, and they found that the MOB1A (Mps one binder kinase activator 1A) gene was modulated downstream of miR-664a-3p through the inactivation of the Hippo signaling pathway. Thereafter, researchers used the JASPAR database and the UCSC genome browser tool and speculated that FOXP3 might be an upstream regulator gene for miR-664a-3p. Correspondingly, they confirmed that FOXP3 could bind to the putative binding site upstream of miR-664a-3p, using a ChIP assay. These data suggest that FOXP3 can promote tumor metastasis in GC (Table 2).

Meanwhile, other studies have found that FOXP3 plays a role in inhibiting metastasis in GC (Table 1). According to Hao et al. [24], FOXP3 can inhibit the activity of NF-κB, which reduces the expression of COX2 (cytochrome c oxidase subunit 2) and inhibits tumor metastasis. Another study showed that FOXP3 expression was associated with favorable clinicopathological variables and good prognosis in 182 GC patients and that FOXP3 expression in GC tissues was correlated with less lymphatic invasion and a lower T stage, N stage, and recurrence rate. In GC tissues, FOXP3 is mainly located in the cytoplasm or nucleus and is positively related to the expression of P21 [25]. Additionally, Pan et al. [26] utilized quantitative proteomic analysis and found that FOXP3 inhibits GC migration and invasion by combining with the promoter region of antioncogene Caveolin-1 (CAV1).

#### 3.1.4. Colorectal Cancer (CRC)

Current studies on the effect of FOXP3 on tumor metastasis in CRC also show contradictory results. One study that focused on 173 cases of CRC, with respect to the clinical characteristics, showed that FOXP3 was negatively related to lymph-node metastasis [27], but that there was no significant difference with respect to distant metastasis (Table 1). However, Wang et al. [28] found that a high level of FOXP3 was associated with the T stage, liver metastasis, and worse clinical outcomes in CRC patients. According to in vitro and in vivo studies, it was discovered that FOXP3 also displayed metastasis-related properties, including cell migration and proliferation. The researchers then conducted weighted gene co-expression network analysis (WGCNA), RNA-seq analysis, and KEGG pathway enrichment analysis to determine the key FOXP3-associated genes/pathways in candidate CRC liver metastasis. Finally, they conducted metabolomics to verify that FOXP3 upregulated MMP9 expression through S-adenosylmethionine (SMA) metabolism. Moreover, Yang et al. [29] suggested that FOXP3 binds to the promoter and intron regions of ZEB2 (zinc finger E-box binding homeobox 2) and miR-155 simultaneously binds to the 3′-UTR region of wild-type ZEB2. The overexpression of FOXP3 and miR-155 might jointly downregulate ZEB2 expression, which would inhibit invasive and migratory capacities and EMT in CRC cell lines (Table 2).

### 3.2. Breast Cancer (BC)

**Figure 4:**
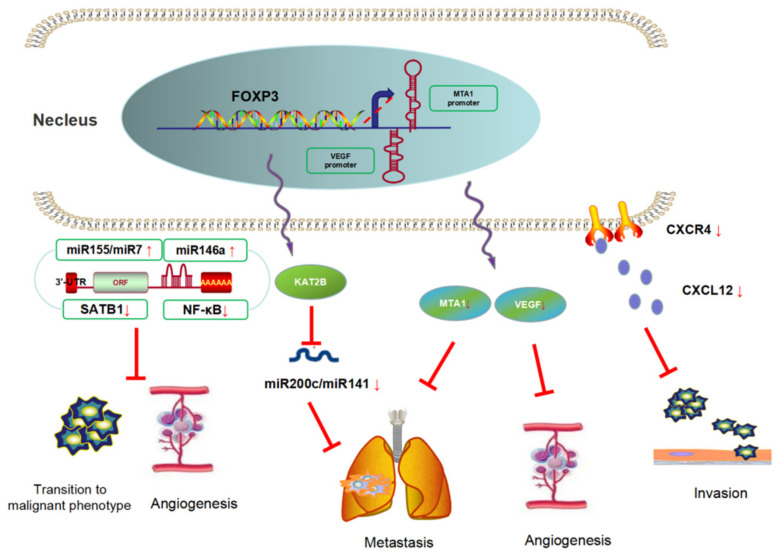
FOXP3 is a suppressor of breast cancer metastasis. FOXP3 could inhibit BC metastasis using three approaches, namely contains angiogenesis genes suppression, chemokines secretion inhibition, and miRNAs negative-feedback loop regulation.

FOXP3 is a suppressor gene in BC metastasis (Figure 4). Li et al. [30] demonstrated that FOXP3 can inhibit the formation of the tube-like structure of human umbilical vein endothelial cells (HUVECs) in vivo and in vitro, and the research then revealed that FOXP3 downregulated the expression of VEGF through the interaction with FKH motifs in the VEGF promoter. Clinical specimen analyses also showed a negative relationship between VEGF and nuclear FOXP3 expression. A similar pathomechanism was found by Liu et al. [31], they revealed that FOXP3 can bind to the MTA1 (metastasis-associated 1) promoter and downregulate the transcriptional activity of the MTA1 gene. MTA1 influences local invasion and lymph-node metastasis and regulates downstream gene transcription activity. They also showed that the FOXP3–MTA1 pathway can reduce the ability of tumor cells to metastasize to the lungs in vivo.

FOXP3 can also affect tumor metastasis by modulating chemokine responses in BC. In invasive BC cell lines, FOXP3 was located predominately in the cytoplasm and significantly lower than that in normal epithelium. Furthermore, FOXP3 expression was negatively correlated with HER2/ErbB2, SKP2 (S-phase kinase-associated protein 2), and CXCR4 (C-X-C motif receptor 4) and positively correlated with CDKN1A (cyclin-dependent kinase inhibitor 1A, p21) in breast cell lines. FOXP3 can also reduce the chemotaxis of CXCL12 (C-X-C motif ligand 12) by inhibiting the expression of CXCR4, which leads to the inhibition of the distant metastases of tumors, and participates in the inhibition of tumor cell adhesion and invasion [32].

Moreover, some studies have focused on the effect of FOXP3 in angiogenesis mediated by regulating microRNAs. It was reported that FOXP3 activated the miR-146a promoter by regulating a miR-146a/NF-κB negative-feedback loop, which led to proapoptotic effects and the inhibition of angiogenesis [33]. McInnes et al. [34] found that FOXP3 induced the expression of miR-155 and miR-7, both of which can directly bind to the 3′-UTR of SATB1 (special AT-rich sequence binding protein 1) and downregulate its expression; thereby, FOXP3 prevented the transition of a normal breast epithelium to a malignant phenotype. Moreover, Zhang et al. [35] identified a FOXP3–KAT2B–miR-200c/miR-141 transcriptional axis in tumor cell lines. In BC patients, high levels of plasma miR-200c and miR-141 were related to tumor metastasis. In a mechanistic investigation using a Foxp3^sf/+^ spontaneous BC mouse model, miR-200c/miR-141 was downregulated in primary breast cancer cells, especially in those of mice with lung metastases. In addition, TCGA database analysis showed that the level of microRNA-200c/miR-141 was regulated by the FOXP3–KAT2B axis, and this conclusion was confirmed in vitro and in vivo. This study suggests that FOXP3 can be used as a potential biomarker for BC metastasis.

### 3.3. Non-Small-Cell Lung Cancer (NSCLC)

**Figure 5:**
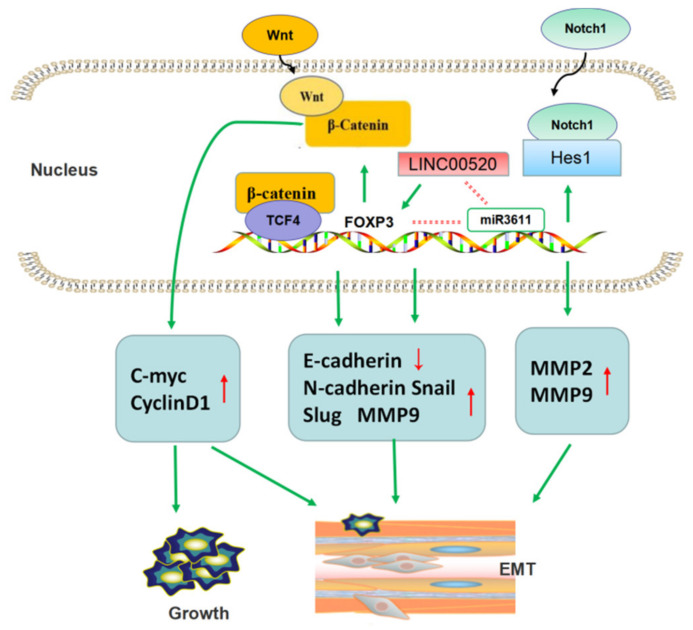
FOXP3 promotes tumor metastasis by promoting EMT in NSCLC. FOXP3 participated in Wnt/β-catenin pathway and Notch1/Hes1 pathway to regulate expression of EMT-related proteins. Furthermore, long non-coding RNA could also promote EMT by activating FOXP3 transcription.

FOXP3 promotes tumor metastasis by promoting EMT in NSCLC (Figure 5). The progression of EMT destroys the normal epithelial structure and leads to tumor cell migration, invasion, and lymphatic vessel invasion, regarded as one of the necessary conditions for tumor invasion and metastasis [36]. Yang et al. [37] found, through clinical trials, that patients with high level of FOXP3 showed obviously decreased OS and RFS. In vitro studies revealed that the ectopic expression of FOXP3 can downregulate E-cadherin and upregulate N-cadherin, Snail, Slug, and MMP9. Further studies confirmed that FOXP3 can induce the transcription of the gene cyclinD1, which modulates the Wnt/β-catenin signal pathway and c-Myc. At the same time, FOXP3 can co-activate the formation and function of β-catenin and the TCF4 (transcription factor 4) complex in the nuclei of tumor cells. These all reciprocally promote tumor invasion and EMT, respectively. Moreover, in vivo studies, a subcutaneous xenograft tumor mice model and a tail-vein-injection metastatic mice model using tumor cell lines with lentivirus-mediated knockdown of FOXP3 were established, and the data showed that FOXP3 could facilitate metastasis of tumor cells to the lung, but not the liver or spleen. Changes in the related signaling pathways have been further confirmed.

In addition, Li et al. [38] uncovered additional signaling pathways by which FOXP3 promotes tumor EMT and metastasis. They found that FOXP3 was overexpressed in NSCLC. FOXP3 can promote the invasion and metastasis of NSCLC cell lines by participating in the regulation of EMT, VEGF, and Notch1/Hes1 signaling pathways. This study also suggested that FOXP3 can upregulate the expression of MMP2 and MMP9, both of which play a promoting role in tumor metastasis. This team also revealed a positive correlation between tumor-derived FOXP3 and lung adenocarcinoma TNM stage, and FOXP3 could inhibit the chemosensitivity to cisplatin. They suggested that FOXP3 might be considered a cisplatin resistance gene of lung adenocarcinoma [39].

Additionally, there was further research focused on the regulation of FOXP3 in lung adenocarcinoma. They found that increased expression of long non-coding (Lnc) RNA LINC00520 indicate poor prognosis in lung adenocarcinoma patients and promoted tumor migration by regulating EMT-related markers E-cadherin and N-cadherin. Research on the mechanism found that FOXP3 induce LINC00520 upregulation by binding to its promoter, and in turn, LINC00520 could function as competing endogenous RNA (ceRNA) against miR3611 to restrain the degradation of FOXP3 [40].

In conclusion, these studies show that a high level of FOXP3 in NSCLC could play a vital role by participating in different signaling pathways, ultimately resulting in tumor EMT. NSCLC can be subdivided into lung adenocarcinoma, SCC, large cell cancer, adenosquamous carcinoma, and others. Adenocarcinoma and SCC are the most common types of NSCLC, and the above-mentioned research was mainly focused on these two types, although there seems to be no difference in the effect of FOXP3 on the two types of cancers.

### 3.4. Squamous-Cell Carcinoma (SCC)

In cervical SCC, Liu et al. [41] suggested that FOXP3 and a newfound LncRNA LINC00885 were highly expressed in cervical SCC. They found that LINC00885 could promote proliferation, invasion, and EMT (upregulating Vimentin and downregulating E-cadherin). Further studies found that FOXP3 is a transcription factor of LINC00885 by dual-luciferase reporter and ChIP assays, which can regulate the IncRNA directly. Researchers considered that FOXP3 can be used as a biomarker of early diagnosis and potential molecularly targeted agents of cervical SCC. In addition, Tang et al. [42] revealed that FOXP3 promote lymph-node metastasis in cervical cancer, and found that the expression of FOXP3 is positively correlated with VEGF-C. However, this research did not perform a mechanism study.

In esophageal SCC, according to Wang et al. [43], the overexpression of FOXP3 was significantly correlated with the tumor TNM stage. Cox-regressive analysis indicated that the T stage, N stage, and level of FOXP3 expression were independent prognostic risk factors in esophageal SCC. Therefore, the research suggests that a high level of FOXP3 is a poor prognosis factor in esophageal SCC.

In oral SCC, the expression of FOXP3 was positively related to lymph-node metastasis. Single-factor analysis and multivariate analysis showed that FOXP3 was an independent prognostic biomarker for five-year overall survival (OS) and recurrence-free survival (RFS) [44] (Table 3).

### 3.5. Melanoma

Skarmoutsou et al. [45] identified that FOXP3 could promote metastasis in melanoma cell lines. Their research compared the expression and subcellular localization of FOXP3 in primary and metastatic melanoma cell lines and normal melanocytes. More importantly, it suggested that the expression of FOXP3 in melanoma metastatic cell lines was markedly highly than that in other cell lines and was mainly localized in the nucleus. This indicated that FOXP3 could be considered a potential independent biomarker of tumor aggressiveness and metastasis. Subsequent research on the mechanism found that NOTCH1 regulate FOXP3 expression via two mechanisms: The direct regulation of FOXP3 transcription and cooperative interaction with the TGF-β1 pathway. However, this study has not been verified by in vivo experiments.

### 3.6. Papillary Thyroid Carcinoma (PTC)

It was reported that FOXP3 was highly expressed in the cytoplasm of PTC tumor cells. A clinical study involving 105 PTC patients found that FOXP3 expression was correlated with distant metastasis, extrathyroid invasion, and poor survival rates. Besides, the researchers found that FOXP3 expression affect radioiodine treatment resistance. In this research, they proposed that FOXP3 could be used as a biomarker for evaluating prognosis [46]. 

### 3.7. Ovarian Cancer (OC)

Zhang et al. [47] indicated that the expression of miR-150-5p and miR-150-3p in OC was lower than that in normal ovarian tissues through GEO datasets analysis. Meanwhile, the research found that miR-150-5p/3p can inhibit tumor migration and invasion both in vitro and in vivo by regulating insulin receptor substrate (IRS) 1 and the Insulin-like Growth Factor 1 Receptor (IGF1R). Further mechanistic research found that FOXP3 can bind to the promoter of miR-150-5p/3p and activate the expression of these miRNAs. Besides, FOXP3-miR-150-IRS1/IGF1R were possibly negative feedback regulated by the PI3K/AKT/mTOR pathway. 

## 4. FOXP3 Interacting with Traditional Chinese Medicine

Traditional Chinese medicine (TCM) is a treasure of Chinese culture, including plants, animals and/or their excreta, minerals, and others. The active constituents of TCM mainly include polysaccharides, flavonoids, saponins, terpenes, and others [48]. TCM plays a significant role in treating many diseases, such as digestive system diseases [49,50], endocrine system diseases [51,52] and autoimmune diseases [53,54]. For the past decades, with the development of researches in cancer treatment, TCM and its active ingredients have shown a satisfactory effect as an adjunctive therapy [55,56,57], and could play an anti-cancer role by modulating immunometabolism [58,59] and improving chemotherapeutic drug resistance [60,61]. Moreover, TCM ingredients can improve anti-tumor immunity and inhibit tumor cell growth and metastasis by reducing the number and function of Tregs and immunosuppressive cytokines secretion.

### 4.1. Immunoregulatory Effect

FOXP3, as a major transcription factor involved in the development and function of Tregs, can induce tumor immune escape in the tumor microenvironment (TME) [62]. Recent studies have revealed that the amino acid sequence encoded by exon 2 of FOXP3 plays an important role in maintaining the lineage stability of Tregs, thus maintaining immune homeostasis and preventing autoimmunity [63].

**Figure 6:**
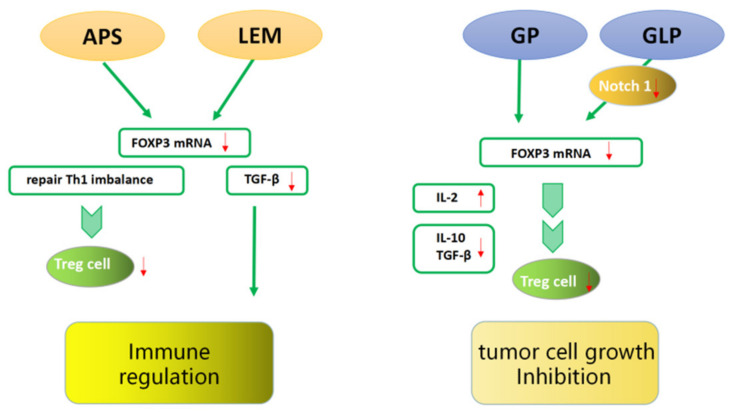
FOXP3 interacting with traditional Chinese medicine. TCM ingredients can improve anti-tumor immunity and inhibit tumor cell growth by reducing the number and inhibiting the function of FOXP3^+^ Tregs and immunosuppressive cytokines secretion.

Li et al. [64] detected the expression of FOXP3 mRNA in the supernatants of HCC tissues and benign tissues. In vitro studies showed that purified astragalus polysaccharides (APS) could significantly reduce the level of FOXP3 mRNA expressed in the supernatant of CD4^+^CD25^+^ Tregs in a dose- and time-dependent manner, which may be related to the reconstitution of cytokines balance and the reduction of FOXP3 expression in TME. Meanwhile, APS can correct the imbalance of Th1 cytokines, inhibit the expression of FOXP3 mRNA, and inhibit Tregs. The role of APS in TME may enhance the efficacy of immunotherapy, thereby improving the survival rate of patients with liver cancer. In another clinical study, the effect of Lentinula edodes mycelia (LEM) therapy on dendritic-cell-based cancer vaccine therapy or CD3-activated T lymphocyte (CAT) therapy was evaluated in 10 cancer patients [65]. The results suggested that the combination treatment of LEM and immunotherapy might increase the expression of FOXP3, CD4^+^, and TGF-β and improve the quality of life and immune function of cancer patients (Figure 6). 

### 4.2. Inhibition of Tumor Cell Growth

The efficacy of TCM in inhibiting tumor growth is also remarkable [66]. He et al. [67] found that the tumor weights of tumor-bearing mice in a purified glycyrrhiza polysaccharide (GP) treatment group were significantly decreased. The expression of FOXP3 and IL-10 mRNA was obviously decreased in the tumor-bearing mice of the treatment group, while the secretion of IL-12 was increased and the contents of IL-10 and TGF-β were decreased. GP may reduce the expression of FOXP3 and upregulate the ratio of Th1/Th2 in the serum, reducing the ratio of Tregs in the TME of H22 tumor-bearing mice and thus inhibiting tumor growth. Li et al. [68] found that a purified *Ganoderma lucidum* polysaccharide (GLP) treatment effectively suppressed the weights of tumors in a dose-dependent manner. The mechanism may be that GLP downregulates the expression of Notch1 through miR-125b to inhibit the expression of FOXP3 and eliminate the Tregs suppression of effector T cell (Teffs) proliferation by increasing IL-2 secretion (Figure 6).

### 4.3. Inhibition of Tumor Cell Metastasis

TCM can also inhibit tumor metastasis effectively [69]. It has been reported that β-Elemene can inhibit tumor angiogenesis and metastasis [70]. Researchers showed that β-Elemene can suppress angiogenesis by inhibiting EMT, which involves molecules such as N-cadherin, E-cadherin, and vimentin. Besides, β-Elemene can block TGF-β1-induced EMT. Given that some studies have reported that FOXP3 can regulate the expression of VEGF and other molecules related to angiogenesis, it is reasonable to believe that FOXP3 can participate in the regulation of tumor metastasis by TCM. The mechanism may be the inhibition of angiogenesis in tumor progression and the inhibition of tumor migration by reducing vascular permeability. Besides, Khinsar et al. [71] found that tumor-derived FOXP3 expression in an H22 transplantation model was decreased after pleurotus ostreatus polysaccharide treatment. Furthermore, anti-tumor cytokines IL-2, Interferon (INF)-γ, and tumor necrosis factor (TNF)α increased. Meanwhile, in vitro, pleurotus ostreatus polysaccharide could inhibit HCC cell line migration, invasion, and metastasis. However, in view of different FOXP3 isoforms playing inconsistent roles in HCC, this study did not identify specific isoforms, and the related mechanism was not clear either.

## 5. Limitations and Deficiencies

There are still imperfections in the current research. The number of clinical research samples is still limited, and the impact of related molecules/pathways on tumor metastasis remains to be explored. Along with this, most of the studies only focused on the FOXP3 gene as an upstream regulator affecting tumor metastasis. The upstream regulatory genes, the related signaling pathways, and the epigenetic changes of FOXP3 in tumors have not been fully elucidated. Meanwhile, both FOXP3^+^ Tregs and tumor-derived FOXP3 are expressed in TME [72], due to the complexity between the cross-talk of FOXP3^+^ Tregs and FOXP3^+^ tumor cells, and it is necessary to further study the performance of communication mechanisms and their effect on tumor biological behaviors.

In addition, FOXP3 expression in different tumors and its influence on metastasis are inconsistent, and intervention strategies need to be formulated according to the corresponding molecular mechanisms. However, knocking out FOXP3 will cause severe autoimmune diseases or spontaneous breast cancer, leading to a lack of neutralizing antibodies or drugs that specifically block tumor-derived FOXP3. Therapy directly targeting FOXP3 is not feasible. Hence, researchers should try to block or activate the downstream target molecules of FOXP3 to intervene with tumor metastasis for clinical treatment.

## 6. Conclusions

There is a growing body of evidence that the FOXP3 gene plays a vital role in tumor metastasis, but the reasons for these discrepant results are still unclear. Several factors may need to be considered. Firstly, the subcellular localization of FOXP3 may contribute to diverse results. Secondly, different kinds of FOXP3 isoforms may function differently in tumor metastasis. What is more important, a complex TME may also affect the role of FOXP3 in tumor metastasis. 

TCM has shown a satisfactory curative effect on malignant tumors due to its low-toxicity side effect. Current studies on the bioactive ingredients of TCM mainly concentrate on the regulation of the body’s immunological function, resistance to chemotherapeutic drugs, and coordination of immune checkpoint blockade (ICB) therapy. Furthermore, there is emerging evidence that TCM has an interaction with the gut microbiome during tumor treatment [73]. On the one hand, the imbalance of the gut microbiome might affect tumor development [74] and is known as a potential biomarker for early diagnosis and prognosis for tumors [75]; on the other hand, anticancer drugs were mediated by the microbiome, especially in immunotherapy [76]. These suggest that researchers could focus on the composition change of the gut microbiome in patients and provide a scientific basis for formulating plans for personal treatment.

In the future, researchers can take advantage of molecular pathologies and bioinformatics to study the relationship between FOXP3 and other molecules in TME, in order to clarify and define the epigenetic changes and regulatory genes related to the FOXP3 gene. Besides, researchers might use TCM for better tumor treatment. In summary, the FOXP3 gene will prove to be a useful target that can bring about new ideas for tumor-targeted therapy.

## Figures and Tables

**Table 1:** FOXP3 acts as a suppressor in digestive system tumors.

Tumor Type	FOXP3 Target	Biological Effects	References
HCC	-	tumor metastasis	Gong et al. [16]
GC	NF-κB inhibition COX2 reduction	tumor metastasis	Hao et al. [17]
GC	P21 induction	lymphatic invasion, T stage, N stage, recurrence rate	Won KY et al. [18]
GC	CAV1 activation	migration, invasion	Pan et al. [19]
CRC	-	lymph-node metastasis	Sun et al. [20]

**Table 2:** FOXP3 acts as a promoter in digestive system tumors.

Tumor Type	FOXP3 Target	Biological Effects	References
HCC	TGF-β/Smad2/3 Pathway enhancement	metastatic potential of tumor cells	Shi et al. [21]
HCC	MMP1 upregulation	non-vascular infiltration, TNM stage, metastasis	Zhang et al. [22]
CCA	MMP2 and MMP9 upregulation	lymph-node metastasis	Ma et al. [23]
GC	TGF-β secretion	tumor migration, invasion, proliferation	Zhang et al. [24]
GC	Activation of WFDC21P	proliferation, lung metastasis, invasion	Cui et al. [25]
GC	-	TNM stage, perineural invasion	Abd-Allah et al. [26]
GC	miR-664a-3p/MOB1A induction, Hippo pathway inactivation	invasion, migration, EMT	Wang et al. [27]
CRC	MMP9 upregulation, SMA metabolism	T stage, liver metastasis, worse clinical outcomes	Wang et al. [28]
CRC	miR-155 induction, ZEB2 reduction	invasion, migration, EMT	Yang et al. [29]

**Table 3:** FOXP3 in SCC.

Tumor Type	Biological Effects	References
Cervical SCC	proliferation, invasion, EMT	Liu et al. [41]
Cervical SCC	lymph-node metastasis	Tang et al. [42]
Esophageal SCC	T stage, N stage, lymph-node metastasis	Wang et al. [43]
Oral SCC	lymph-node metastasis, OS, RFS	Song et al. [44]
